# Autonomous fetal morphology scan: deep learning + clustering merger – the second pair of eyes behind the doctor

**DOI:** 10.1186/s12911-024-02505-3

**Published:** 2024-04-19

**Authors:** Smaranda Belciug

**Affiliations:** Department of Computer Science, Faculty of Sciences, University of Craiova, 200585 Craiova, Romania

**Keywords:** Deep learning, Clustering, Segmentation, Fetal morphology

## Abstract

The main cause of fetal death, of infant morbidity or mortality during childhood years is attributed to congenital anomalies. They can be detected through a fetal morphology scan. An experienced sonographer (with more than 2000 performed scans) has the detection rate of congenital anomalies around 52%. The rates go down in the case of a junior sonographer, that has the detection rate of 32.5%. One viable solution to improve these performances is to use Artificial Intelligence. The first step in a fetal morphology scan is represented by the differentiation process between the view planes of the fetus, followed by a segmentation of the internal organs in each view plane. This study presents an Artificial Intelligence empowered decision support system that can label anatomical organs using a merger between deep learning and clustering techniques, followed by an organ segmentation with YOLO8. Our framework was tested on a fetal morphology image dataset that regards the fetal abdomen. The experimental results show that the system can correctly label the view plane and the corresponding organs on real-time ultrasound movies.

**Trial registration**

The study is registered under the name “Pattern recognition and Anomaly Detection in fetal morphology using Deep Learning and Statistical Learning (PARADISE)”, project number 101PCE/2022, project code PN-III-P4-PCE-2021–0057. Trial registration: ClinicalTrials.gov, unique identifying number NCT05738954, date of registration 02.11.2023.

## Introduction

Congenital abnormalities or disorders can be structural or functional, and they occur during intrauterine life. Some of these disorders are detected prenatally during the second trimester morphology ultrasound scan, at birth, or later in infancy. According to the World Health Organization, each year, around 240 000 newborns die in their first month after birth, due to birth defects (https://www.who.int). 170 000 die before their 5th birthday. The occurrence of congenital abnormalities is high in low and middle-income countries, due to social, economic, and racial reasons [1]. The deficiency of nutritious food, increased exposure to infections, alcohol, lack of prenatal care, and screening lead to a higher risk of birth defects. Detecting abnormalities early on can facilitate potential life-saving treatments, or manage certain disabilities, by stopping their progression. Birth defects can be diagnosed prenatally by performing a morphology scan [2]. Current methodologies fail to achieve high performance in detecting abnormalities. There have been reported disagreements between pre- and post-natal diagnosis that range between 27.5% and 96% in terms of sensitivity [3]. Different studies present different accuracies in detecting congenital abnormalities during the morphology scan, from 32.5% (unexperienced sonographer) to 52% (experienced sonographer) [4]. A recent study, conducted in a Danish region, reported a detection rate of 69% [5].

The first step towards detecting congenital anomalies is detecting the organs automatically during a morphology scan. Some types of birth defects are represented by the non-visualization of the specific organ (e.g. stomach, nose, limbs, corpus callosum, bladder, gallbladder, etc.) [6–8]. Therefore, if an autonomous intelligent system is not able to find in an ultrasound movie a certain organ, that implies that the fetus might suffer from a congenital anomaly. Even though this subject is critical and sensitive, relatively little work has been published regarding this domain. In two studies that regard the fetal heart, conducted by the Department of Obstetrics and Gynecology Showa University School of Medicine, Fujitsu, and the Cancer Translational Research Team, the researchers report a recall that ranged between 61.9 and 100 [9, 10]. In [11], the 3D fetal brain has been segmented and reconstructed using convolutional neural networks. For the fetal brain and lungs segmentations, the researchers obtained a dice score of 0.78, after applying DL, sequential forward feature selection techniques, and Support Vector Machines [12], while for the fetal head, after applying only DL, the reported dice score ranged between 0.70 and 0.76 [13]. A mixture between convolutional neural networks and Differential evolution was applied on two maternal fetal datasets achieving 96.20% and 78.73% accuracy, respectively [14]. A merger between DL and evolutionary computation achieved 74.62% accuracy when applied on a dataset regarding the fetal abdomen. Reached a 74.62% accuracy [15]. In [16], DL together with Gaussian Mixture Modelling obtained: 81.92% and 92.12% accuracy, on two maternal fetal datasets. For further reading regarding the fetal ultrasound segmentation, we mention [17–19].

This study is intended to provide a solution for early detection of congenital anomalies using Artificial Intelligence (AI). Therefore, our aim is to build an AI-powered decision support system based on a synergetic merger between different DL algorithms that perform feature extraction with transfer learning from 2D ultrasound images, which are further on clustered using different clustering methods to differentiate the view planes of the abdomen of a second trimester fetal morphology scan. During a morphology scan, as the fetus moves and breathes, images that correspond to a view plane are mixed with images that correspond to other view planes of the abdomen. We are using clustering techniques so we could group together the images that represent the same view plane. After the clustering process is over, we use YOLO8 to segment the anatomical structures in each image, to help the doctors detect easier potential abnormalities.

The paper is organized as follows: in Sect. "Materials and methods", we will present the design and implementation of the proposed AI-powered decision support system, the dataset used, and the design of experiments and used parameters. The results and their statistical analysis are discussed in Sect. "The framework". Sect. "DL Competitors" deals with the discussion, while Sect. "Clustering competitors" covers the conclusions and future work.

## Materials and methods

### The framework

The AI-powered decision support system is built on four pillars: DLs for feature extraction from ultrasound images, clustering methods for clustering the obtained information, YOLO8 for segmenting the organs, and statistical analysis to validate our findings. Since the DLs and the clustering methods are of stochastic nature, the only way to determine whether the obtained results are effective, as well as robust, is to independently run all the AI algorithms a certain number of times in a complete tenfold cross-validation. Therefore, we have performed power analysis to guarantee that the statistical tests applied have achieved adequate power. The sample size estimation method involved the two-tailed type of null hypothesis, having a default statistical power goal $P\ge 95\%$, with type I error. For each DL method we have used the standard tenfold cross-validation. In this way, we have eliminated the possibility of overlearning. The performance of each algorithm was computed as the percentage of correctly classified cases.

To choose the best performing DL algorithm, we have recorded the accuracy obtained in the testing phase and the corresponding standard deviation (SD). This step was followed by a thorough statistical analysis of the DL’s performances, involving *data screening* (i.e. normality tests such as Anderson–Darling and Jarque–Bera tests, and equality of variances tests such as Levene’s and Brown-Forsythe tests), and one-way ANOVA test which comprised the sum of squares (SS), mean squares (MS), degrees of freedom (df), *F*-value, and *p*-value. The one-way ANOVA was followed-up by the post-hoc Tukey test. These follow-up tests revealed which DLs are statistically significant different in terms of performance. Taking into account the “no-free-lunch” theorem, we cannot state that there is only one best performing DL for feature extraction, their performance being dependent on the dataset they have been applied on. Hence, we have chosen heuristically five DL methods, and statistically compared their performances, to find the best suited one for this dataset. Future work will include statistically analyzing the performance of other DLs.

Using only the best performing DL, we extract features from the ultrasound images. Feature extraction transforms raw data (images) into numerical features, so we can further process them while keeping the information in the original database. Each layer from the DL extracts one or multiple unique features from an image. We define a feature as any part or pattern of an object used to identify it in an image. Features can be corners, regions of interest points, ridges, edges, blobs of color, etc. In our study, we have used discriminative filters learned by state-of-the-art DLs on ImageNet, after which we have applied these DL networks to recognize objects on a second trimester morphology scan dataset.

**Fig. 1:**
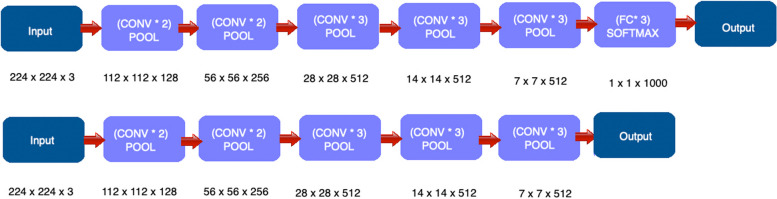
VGG-16 network architecture for classification vs.VGG-16 network architecture for feature extraction

When we are performing feature extraction, we are mainly using the pre-trained DL networks as an arbitrary feature extractor, that allows the input image to propagate forward throughout the network, only to stop at a predefined layer. The features will be the outputs of that layer. For a better understanding of the process, we illustrate in Fig. 1, how a certain type of DL, called VGG16, extracts features from an image.

To obtain the feature vector, we allow the image to propagate throughout the network, and we stop this process prior to the fully connected layer. If we stop the propagation process at this step, we obtain an output that has the shape of $7\times 7\times 512$. The feature vector is extracted by flattening this volume to a list of numbers that will quantify our input image, $7\times 7\times 512=25088$. If we have *n* images in the training dataset, then after the feature extraction process is over, we will have *n* vectors, each one of them having a 25,088 dimension. Each clustering technique groups these features.

**Fig. 2:**
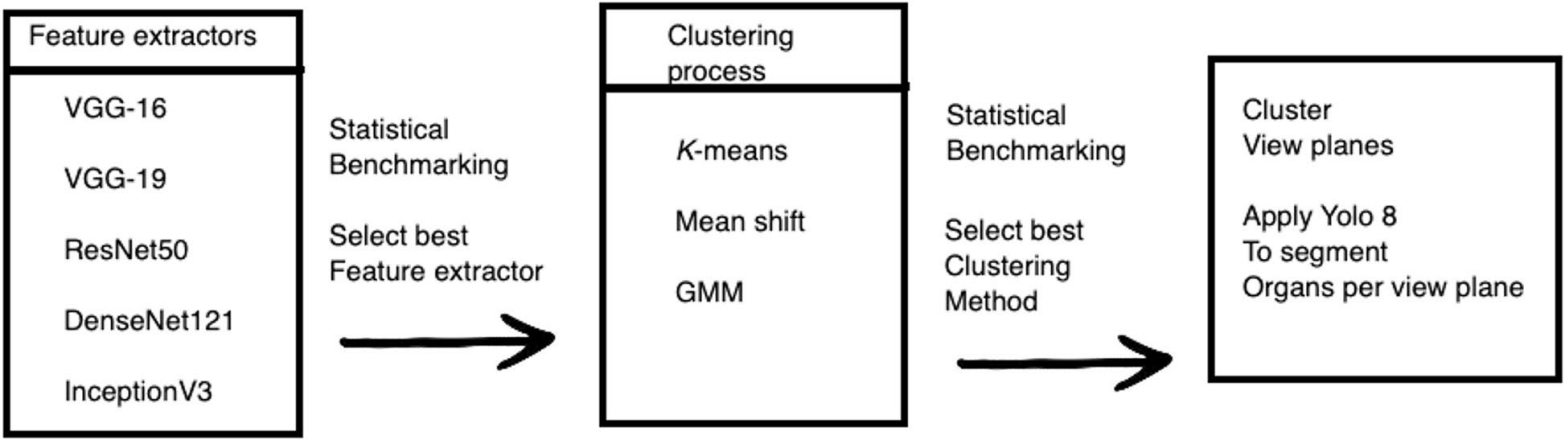
Overview of the proposed framework’s architecture

We record for each clustering method the decision performance on the testing data, and its standard deviation. Another statistical analysis similar to the one presented above is performed on the recorded results. The best performing DL together with best clustering technique are kept for standard view plane differentiation. After this step is over, we apply YOLO8 to segment the anatomical organs in each view plane (Fig. 2).

#### DL Competitors

In general, when we are dealing with DL neural networks, we encounter in their architecture three types of layers: the convolutional layer, the pooling layer, and the fully connected layer. Since we are using the DLs for feature extraction purposes, we will remove from their architecture the last layer, the fully connected one. The feature map is computed by convolution operations that use a filter to scan the input. The filter is characterized by its size and stride. Another parameter encountered in a DL is the zero-padding. As we can see in Fig. 1, one or multiple convolutions are followed by a pooling layer. The role of the pooling layer is to down sample the feature map and to produce spatial invariance.

In general, the activation function used in a DL is the rectified linear unit function (ReLU). However, in literature we find other variants like Leaky ReLU, Exponential linear unit (ELU), and softmax. ReLU, leaky ReLU, and ELU produce non-linearities, while softmax transforms the input score vector into a probability vector. In mathematical terms, we can express these functions as follows:

- ReLU:

$$f\left(x\right)= \left\{\begin{array}{c}0, x<0\\ x, x\ge 0\end{array}.\right.$$

- Leaky ReLU:

$$f\left(x\right)= \left\{\begin{array}{c}0.01\cdot x, x<0\\ x, x\ge 0\end{array}.\right.$$

- ELU:

$$f\left(a, x\right)= \left\{\begin{array}{c}a\cdot \left({e}^{x}-1\right), x<0\\ x, x \ge 0\end{array}\right.$$

- Softmax:

$$p= \left(\begin{array}{c}{p}_{1}\\ \begin{array}{c}.\\ .\end{array}\\ {p}_{n}\end{array}\right), \mathrm{where }{p}_{i}= \frac{{e}^{{x}_{i}}}{\sum_{j=1}^{n}{e}^{{x}_{j}}}.$$

In this study we have used the following DL networks:

- VGG-16 won the 1st and 2nd place in 2014 ISLVR challenge. It has been developed by the Visual Geometry Group Lab of Oxford University [20]. VGG-16 has 13 convolutional layers, 5 max pooling, and 3 dense layers. This type of DL network focuses in having convolution layers of $3\times 3$ filters with stride 1, and the max pooling layer of $2\times 2$ filters with stride 2. For feature extraction we remove the fully connected layer.
- VGG-19 is a variant of the VGG model that has 16 convolutional layers, 3 fully connected, 5 max pooling layers, and 1 softmax layer. To act as a feature extractor, we remove the last fully connected layer from its architecture.
- ResNet50 (Residual Network 50) won the ILSVR (ImageNet) contest in 2015. ResNet uses a skip connection, that permits the gradient flow to have a complementary cutoff route. The skip connection facilitates the higher-level layers of the model to perform as well as the lower ones. Its architecture contains 48 convolutional layers, 1 maxpool layer, and 1 average pool [21]. To use ResNet50 as a feature extractor, we train it with one-hot encoded labels, and take away the fully connected layer.
- DenseNet121 (Dense CNN) is a variant of ResNet that represents a better solution for the gradient vanishing problem. In this type of network, the propagation of features is strengthened, and the number of parameters is reduced. In a DenseNet121 architecture we have five blocks of batch normal convolutional layers. Between two such blocks there is a transition layer used for merging the preceding feature maps from the same block used as input for the next block. We drop the last fully connected layer from the architecture to extract the feature vector [22].
- InceptionV3 uses factorized convolutions. Big convolutions are replaced with smaller ones, and asymmetric convolutions are added to the architecture. In this way the training process is speeded up. An auxiliary classifier is added to the network. This classifier acts as a regularizer [23]. The last fully connected layer is dropped for feature extraction.

All five DLs have the same classifier, activation function ReLU, optimizer adam. The loss function is the categorical cross entropy.

### Clustering competitors

The feature vectors obtained by the best performing DL network were clustered using the following methods: *k*-means, mean-shift, and Gaussian Mixture Modelling (GMM). The rationale behind choosing these clustering techniques was based on the data type and structure of our data, the number of clusters, and the clusters’ shape and size. For instance, our data is numeric, so we needed to use algorithms that are based on distance measures such as *k*-means or hierarchical clustering, and not *k*-modes, DBSCAN or fuzzy clustering. We decided to drop the hierarchical clustering method due to time complexity and irreversibility. Mean-shift was chosen because it can determine the number of clusters automatically. Because we had no prior knowledge regarding the clusters’ shapes (spherical or elliptic), we decided to use both *k*-means and GMM.

We define a cluster as a set of data points similar to each other. The goal of the clustering procedure is to split the set of data points into a number of clusters (groups) in such a way that the data points within each group resemble one another and differ from the data points in other groups.

### k-means

*K*-means is one of the most popular clustering methods. It uses *k* centroids that define the number of clusters. We assign a data point to a particular cluster, if it is ‘closer’ to that cluster’s centroid than any other centroids. The centroids are at first set arbitrarily, after which they are computed as the center of a cluster. *K*-means clusters data alternating the following steps: a) assigns data points to the nearest centroid; b) computes the new centroids using the currently assigned data points.

Let us denote the training set as $Train= \{{{\text{x}}}^{1},\dots , {{\text{x}}}^{n}\}$, where each ${{\text{x}}}^{j}, j=1,\dots ,n$ is an image feature vector. We need to add the label ${c}^{(i)}$ to each data point, $i=1,\dots , k$. The *k*-means algorithm is as follows:

1. Randomly initialize the cluster centroids ${\mu }_{1}, {\mu }_{2}, \dots , {\mu }_{k}\in {\mathbb{R}}^{l}$, *l* being the number of features.
2. Repeat until convergence:

For every *i,* set the label:

$${c}^{(i)}={\text{arg}\,}{\text{min}_{j}\Vert {{\text{x}}}^{\left\{i\right\}}-{\mu }_{j}\Vert }^{2}.$$

For each *j*, compute the new centroids:

${\mu }_{j}= \frac{1}{\left|{n}_{j}\right|}\sum_{i=1}^{{n}_{j}}{{\text{x}}}^{i}$, where ${n}_{j}$ is the number of data points in cluster *j*.

*K*-means is very sensitive to initialization, which can lead to poor convergence speed and an overall bad clustering performance. Even though it works well for round shaped equal sized clusters, it fails in performance when dealing with non-convex shapes.

### Mean-shift clustering

Mean shift clustering is a non-parametric algorithm which uses density to identify clusters in a dataset. This type of algorithm works well on data that has an arbitrary shape and does not have well-separated linear boundaries. Taking into account a certain radius, the method shifts each data point towards the highest density of the distribution of points, also known as mode. This process is repeated until all the points converge to a local maximum representing the clusters in the data. Unlike *k*-means, in the mean-shift algorithm we do not specify the number of clusters a priori. The algorithm determines by itself the number of clusters. A downside of the algorithm is the fact that it is computationally expensive ($O({n}^{2})$).

Having *N* data points ${{\text{x}}}_{i}\in {\mathbb{R}}^{d}$, *d* being the number of features, we define the kernel density estimate using a radially symmetric kernel, $K\left({\text{x}}\right),$ as:

$${\widehat{f}}_{K}=\frac{1}{N{h}^{d}}\sum_{i=1}^{N}K\left(\frac{{\text{x}}-{{\text{x}}}_{i}}{h}\right),$$

where *h* is the bandwidth parameter that defines the kernel’s radius.

Mathematically speaking, the radially symmetric kernel is computed using the following formula:

$$K\left({\text{x}}\right)= {c}_{k}k\left({\Vert {\text{x}}\Vert }^{2}\right),$$

where ${c}_{k}$ is the normalization constant. The algorithm ends with when it reaches convergence, i.e.$\nabla f\left({x}_{i}\right)=0.$

Using the gradient of the density estimator, we compute:

$$\nabla \widehat{f}\left({\text{x}}\right)= \frac{2{c}_{k,d}}{n{h}^{d+2}}\left[\sum_{i=1}^{n}g\left({\Vert \frac{{\text{x}}-{{\text{x}}}_{i}}{h}\Vert }^{2}\right)\right]\left[\frac{\sum_{i=1}^{n}{{\text{x}}}_{i}g\left({\Vert \frac{{\text{x}}-{{\text{x}}}_{i}}{h}\Vert }^{2}\right)}{\sum_{i=1}^{n}g\left({\Vert \frac{{\text{x}}-{{\text{x}}}_{i}}{h}\Vert }^{2}\right)}-{\text{x}}\right],$$

where $g\left(x\right)=-k{\prime}(x)$ is the derivative of the selected kernel. The first parenthesis of the above formula is proportional to the density estimate at x, having the kernel $G={c}_{g}g\left({\Vert {\text{x}}\Vert }^{2}\right)$. The second parenthesis represents the mean shift vector, *m*, and it points toward the direction of the maximum increase in density. It is proportional to the density gradient estimate at point x, using the kernel *K* [24].

Technically, the mean shift algorithm is summarized as:

1. Compute the mean shift vector $m\left({x}_{i}^{t}\right)$.
2. Shift the data point toward the mean ${x}_{i}^{t+1}={x}_{i}^{t}+ m\left({x}_{i}^{t}\right).$
3. Repeat 1 and 2, until the algorithm reaches convergence, $\nabla f\left({x}_{i}\right)=0.$

### Gaussian mixture modeling

The GMM algorithm presumes that all the data points are governed by the Normal distribution, so the shape of the clusters is described not using only one parameter, the mean, but two parameters: the mean and SD. Since the SD goes both ways, the shape of the cluster is elliptic, and not circular. Each cluster has assigned a Gaussian distribution. The GMM clustering method merges multiple such Gaussian distributions. We compute the probability density function for 1-dimension as:

$$f\left(X|\mu ,\sigma \right)= \frac{1}{\sigma \sqrt{2\pi }}{e}^{\frac{-{(X-\mu )}^{2}}{2{\sigma }^{2}}},$$

where $\mu$ and $\sigma$ are the mean and SD of the distribution. If we are dealing with a multivariate d-variate dimension, then the probability density function will be computed as:

$$f\left(X|\mu ,\Sigma \right)= \frac{1}{\sqrt{2\pi }|\Sigma |}{e}^{\left(-\frac{1}{2}{\left(X-\mu \right)}^{T}{\Sigma }^{-1}(X-\mu )\right)},$$

where $\mu$ is the mean of the distribution, $\Sigma$ is the covariance matrix of *X*, *T* is the transpose of a vector, and -1 the inverse of a matrix.

For each cluster we estimate $\mu$ and $\Sigma$. Since we are not dealing with only one cluster, we cannot estimate these values using the maximum-likelihood method, we need to define the probability density as a linear function of the cluster’s distribution densities:

$$p\left(X\right)= \sum_{k=1}^{K}{\pi }_{k}f(X|{\mu }_{k},{\Sigma }_{k}),$$

where ${\pi }_{k}$ is the mixing coefficient for cluster’s *k* distribution.

We then can compute:

$$p\left(X|\mu ,\Sigma ,\pi \right)=\sum_{i=1}^{N}{\text{ln}}\sum_{k=1}^{K}{\pi }_{k}f({X}_{i}|{\mu }_{k}, {\Sigma }_{k}).$$

making use of the maximum log-likelihood method. Using Bayes’ theorem, we can define a random variable ${\beta }_{k}\left(X\right)=p(k|X)$, and compute as:

$${\beta }_{k}\left(X\right)= \frac{p\left(X|k\right)\cdot {\pi }_{k}}{\sum_{k=1}^{K}{\pi }_{k}p(X|k)}.$$

If the derivative of $p\left(X|\mu ,\Sigma ,\pi \right)=0$ with respect to $\mu$ and $\pi$, then the maximum value of the log-likelihood function is reached, thus the parameters can be estimated by computing:

$${\mu }_{k}=\frac{\sum_{n=1}^{N}{\beta }_{k}({x}_{n}){x}_{n}}{\sum_{n=1}^{N}{\beta }_{k}({x}_{n})},$$

$${\Sigma }_{k}= \frac{\sum_{n=1}^{N}{\beta }_{k}({x}_{n})({x}_{n}-{\mu }_{n}){({x}_{n}-{\mu }_{n})}^{T}}{{\sum }_{n=1}^{N}{\beta }_{k}({x}_{n})},$$

and

$${\pi }_{n}= \frac{1}{N}\sum_{n=1}^{N}{\beta }_{k}\left({x}_{n}\right),$$

where $\sum_{n=1}^{N}{\beta }_{k}\left({x}_{n}\right),$ is the total number of data points in cluster *k*, *n* is the total number of samples ${x}_{i}$.

The *expectation maximization* algorithm initializes ${\mu }_{k}, {\Sigma }_{k},$ and ${\pi }_{k}$ with random values, followed by estimating the latent variables (${\beta }_{k})$, and by updating the parameters’ values. Having estimated the parameters of each Gaussian, we can proceed to cluster the data points.

### Second trimester morphology scan dataset

The proposed AI powered decision support system was applied on a dataset containing second trimester fetal morphology scans. The data comes from a prospective cohort study implemented in a maternity hospital in Romania, University Emergency County Hospital of Craiova. The participants are pregnant patients that have admitted themselves at the Prenatal Unit of the County Hospital for the second trimester morphology scan. Prior to the standard consultation and ultrasound scan, the OB-GYN doctors members of the research team, have informed the patients about the conducted research and invited them to take part of the study. After the patients understood the study’s implication, we obtained their written consent.

**Fig. 3:**
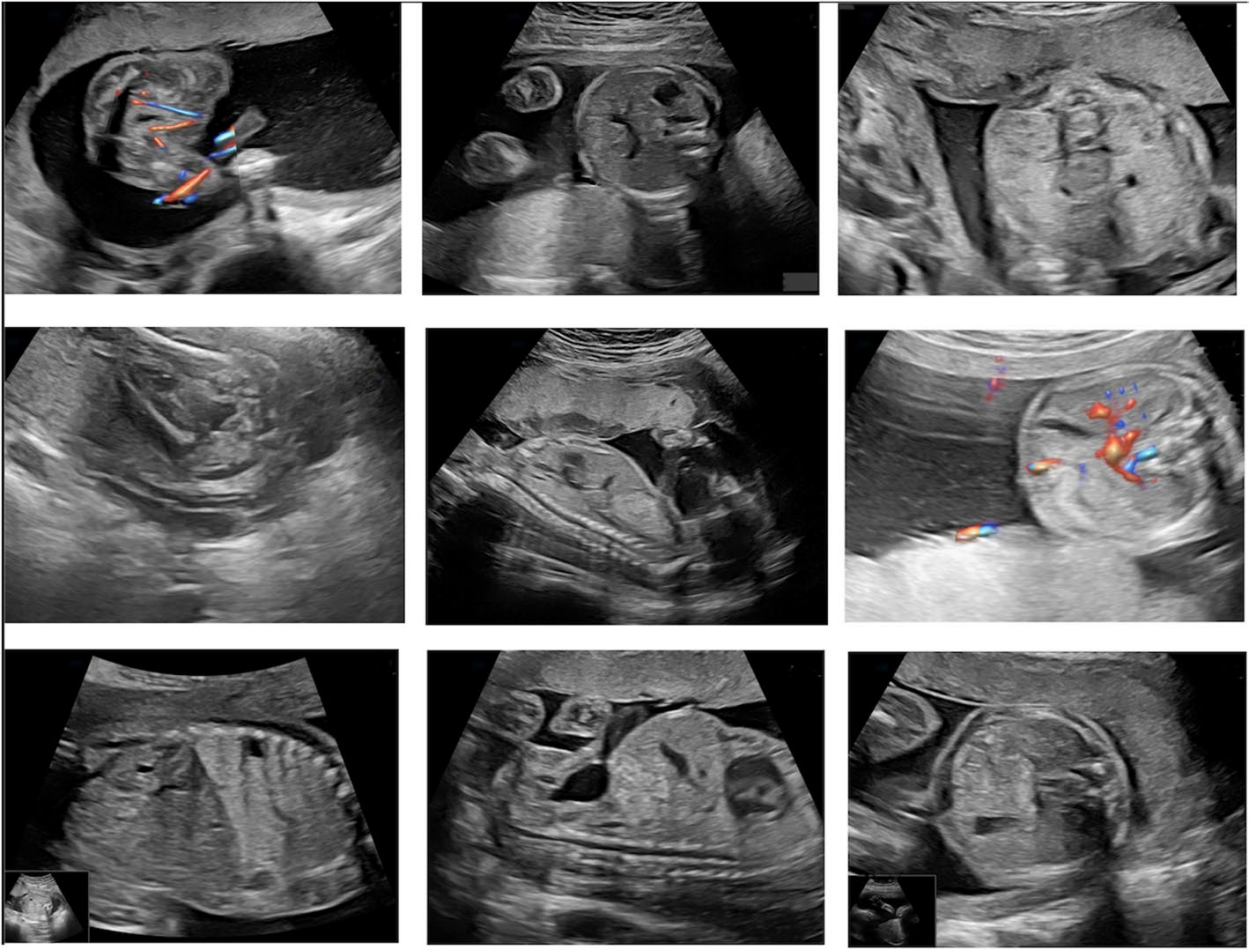
Sample image from each class: 3 vessels plus bladder plane; abdominal circumference plane; anteroposterior kidney plane; bladder plane; echogenic plane; gallbladder plane; longitudinal kidney plane; cord insertion sagittal plane; transabdominal cord insertion plane

The OB-GYN team members have a minimum 2-year experience and are board certified in performing transabdominal obstetrical ultrasound. The images were acquired using Logic e (GE Healthcare, China US machines with 2–5-MHz, 4–8-MHz, and 5–9 MHz curvilinear transducers) and Voluson 730 Pro (GE Medical Systems, Zipf, Austria). The dataset contains 3279 images of the fetal abdomen collected from 215 patients. The images were anonymized and secured. The data was split into 9 decision classes: 3 vessels plus bladder plane (591 images), abdominal circumference plane (421 images), anteroposterior kidney plane (373 images), bladder plane (217 images), echogenic plane (258 images), gallbladder plane (332 images), longitudinal kidney plane (622 images), cord insertion sagittal plane (215 images), transabdominal cord insertion plane (250 images). Figure 3 presents a sample image from each view plane.

**Fig. 4:**
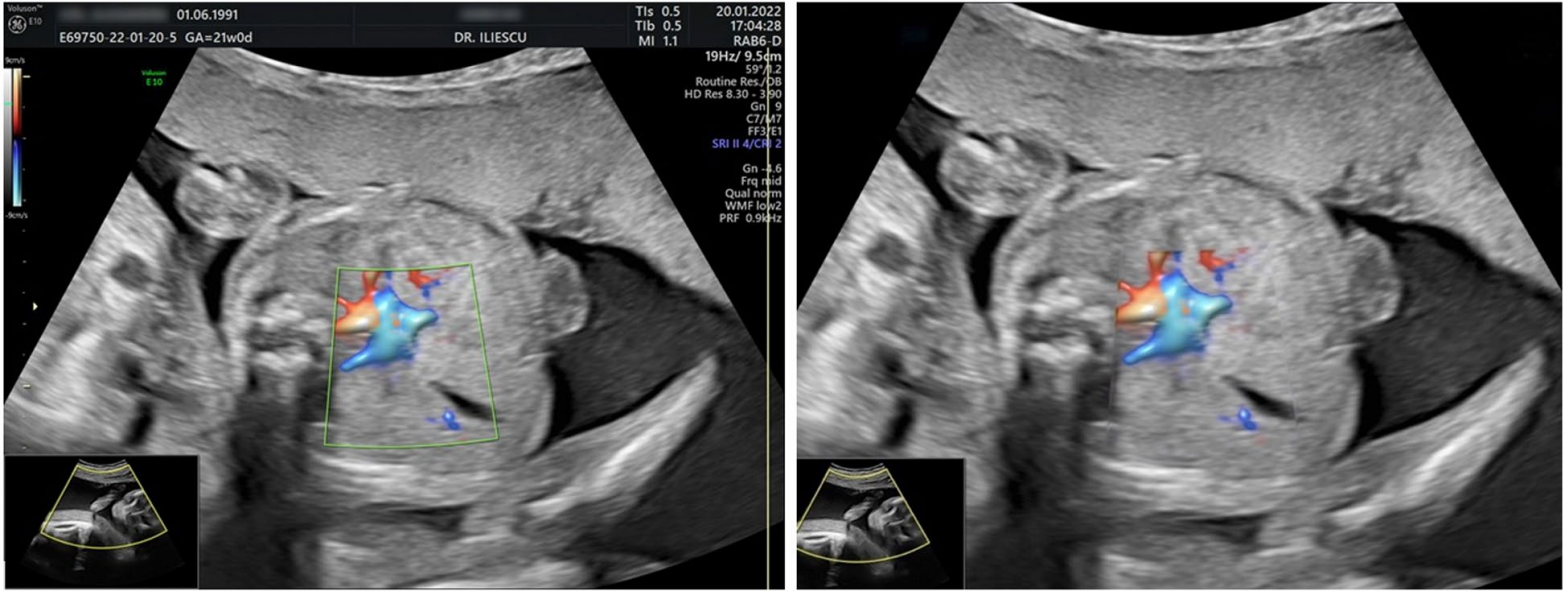
On the left we have an unprocessed image with text and calipers, on the right we have the same image without the text and calipers, which have been removed with CV2 and Keras OCR

As it can be easily seen, the dataset is unbalanced, but this is a common scenario encountered in clinical practice. Since this is an ongoing study, the doctors are recording images from the classes that have fewer samples. All data was processed and artefacts and text were removed. This task was performed using CV2 and Keras-OCR. Using Optical Character Recognition, the text was detected in images and removed. Around each text, the algorithm created a bounding box containing its coordinates. The boxes had masks applied onto, which the algorithm inpainted with the surrounding pixels. Figure 4 shows an image before and after this preprocessing step.

A data generator was used to create more samples in the dataset. In this way, the overfitting effect is avoided. Each image was transformed using shear rage = 0.2, zoom range = 0.2, rotation range = 20, width shift range = 0.1, height shift range = 0.1, brightness range $\in [0.7, 1.4]$. The images were then resized to $224 \times 224$ px. As the project progresses, images are dynamically added to the dataset, as more and more ultrasounds are performed. The version of the dataset is no longer available. Permission on the up-to-date version of the second trimester fetal morphology dataset is available upon request.

Using LabelStudio (https://labelstud.io/), the doctors have labeled each anatomical structure that can be assessed in each view plane. Thus, the following organs have been labeled in each view-plane:

- 3 vessels and bladder view plane: bladder, vessels;
- abdominal circumference plane: aorta, confluence, rib, spine, stomach;
- anteroposterior kidney plane: basin, kidney;
- bladder plane: bladder;
- echogenic plane: intestines;
- gallbladder plane: gallbladder;
- longitudinal kidney plane: kidney;
- cord insertion sagittal plane: intestines, wall_and_cord;
- transabdominal cord insertion plane: wall_and_cord.

## Results

Our study was three-fold: *a*). to find out which DL performs better at extracting features from images and use it to transform the image dataset into feature vector dataset; *b*). to find out which clustering techniques clusters best the feature vectors and use it to classify the data; *c*). to apply YOLO8 to segment the images clustered in the previous step.

YOLO is short for You Only Look Once. YOLO has the ability to perform real-time object detection by just one step through the neural network, making it fast as well as efficient. Traditional CNNs use complex multi-stage pipelines, making the computational load significantly higher. Clustering methods are also computational expensive when dealing with high-dimensional feature spaces. Since, our aim is creating an autonomous tool for the morphology scan that can be used in real-time by the medical professionals, we needed to use a method that offered substantial speed improvement, so we chose YOLO. YOLO is able to detect and localize objects, by providing bounding boxes around the regions of interest (ROI), whereas clustering focuses on grouping pixels with similar properties. Lastly, YOLO is more accurate than clustering since it is a supervised learning technique.

In this section, we will cover the experimental results obtained by the DL competitors when applied to extract features from images, followed by the experimental results obtained by the clustering competitors when applied to cluster the feature vectors extracted by the best DL. We have used the following setting for the experiments: the dataset was divided into training and testing and hold out the images from one patient for testing purposes. This setting was proposed and used in order to avoid potential information leakage.

We have performed power analysis to compute how many independent computer runs are needed to achieve a statistical power of 95% with type I error $\alpha =0.05.$ The power analysis revealed that we need a sample size of 50 independent computer runs, therefore all the DL algorithms have been run for 50 times in a complete tenfold cross-validation cycle. In Table 1 we present the performance results of the 5 DLs in terms on average accuracy (ACA) over 50 computer runs and SD.

**Table 1:** Performance of DL algorithms (average accuracy (ACA) and standard deviation (SD)

Model	ACA	SD
VGG-16	68.23	1.99
VGG-19	74.12	2.03
ResNet50	89.23	1.88
DenseNet121	93.11	1.60
InceptionV3	81.23	1.87

From Table 1, we can see that the best performing DL was DenseNet121, followed by ResNet50 and InceptionV3. VGG16 performed the poorest. Looking at the SD, we can see that even if the performances differ in terms of accuracy, all models are robust, the SD ranging between 1.60 and 2.03.

Since the performance of AI algorithms cannot be compared only by comparing accuracies, we have continued our statistical analysis with the *data screening* process, necessary if we wish to apply one-way ANOVA. The data screening process involved performing *Anderson–Darling* and *Jarque–Bera* tests for verifying the normality of the sample data, and *Levene* and *Brown-Forsythe* tests for verifying the equality of variances. Both Anderson–Darling and Jarque–Bera tests revealed that the 50 independent computer runs samples are normally distributed (*p*-level > 0.05). In terms of the equality of variances, even if in some cases (DenseNet121 vs. VGG16, and DenseNet121 vs. VGG19, ResNet50 vs. VGG16, and ResNet50 vs. VGG19) the tests showed that the samples do not have equal variances (*p*-level < 0.05), we can make use of the fact that we have the same number of observations in our samples (50 independent computer runs) and presume that the variances are equal, and proceed with applying one-way ANOVA and post-hoc Tukey test.

One-way ANOVA test results are: sum of squares = 54,812, degrees of freedom = 4, mean squares = 10,384, *F*-value = 2076.3, and *p*-level = 0.000 (contrast quadratic polynomial) [25]. From these results it is clear that there are significant differences between the DLs’ performances, the only thing remaining to be discovered being between which DL are those differences. The post-hoc Tukey test showed that there are significant statistical differences between all models except DenseNet121 and ResNet50 (*p*-level > 0.05).

Taking into consideration the results of the statistical analysis, we decided to continue our experiment using DenseNet121. The next step in our AI powered decision support system was to cluster the feature vector extracted by DenseNet121 using *k*-means, mean-shift clustering, and GMM. We have used the same sample size, 50 independent computer runs, for building the performance samples of the three clustering methods. The clusters’ performances in terms of ACA and SD are displayed in Table 2.

**Table 2:** Performance of clustering algorithms (average accuracy (ACA) and standard deviation (SD)

Model	ACA	SD
*k*-means	68.93	3.02
Mean-shift	92.95	2.14
GMM	89.56	2.01

From Table 2, we see that the best performing clustering in mean-shift clustering reaching an average accuracy of 92.95%, and a SD of 2.14, proving its robustness. GMM method obtained an average accuracy of 89.56% with a 2.01 SD. *K*-means performed the poorest, having an accuracy below 70%.

The data screening process showed that all performances samples are governed by the Normal distribution, except *k*-means (*p*-level < 0.05). However, because the sample size is large enough (> 30 samples) the distribution tends to have a ‘normal’ shape, even if it is not governed by the Normal distribution [26]. The proof of this assumption can be found in the *Central Limit Theorem,* and also in the *t*-table, where if look at the *t* values for 30 degrees of freedom, we see that they become almost equal to the *z* statistics value.

Having these prerequisites checked, we proceeded with applying one-way ANOVA and post-hoc Tukey. The results of one-way ANOVA are sum of squares = 37,231, degrees of freedom = 2, mean squares = 7251, *F*-value = 627.2, and *p*-level (contrast quadratic polynomial) = 0.000. The post-hoc Tukey revealed that there are significant differences between all models. After performing this statistical analysis, we concluded that the most suited clustering algorithm for this case is mean-shift clustering.

**Fig. 5:**
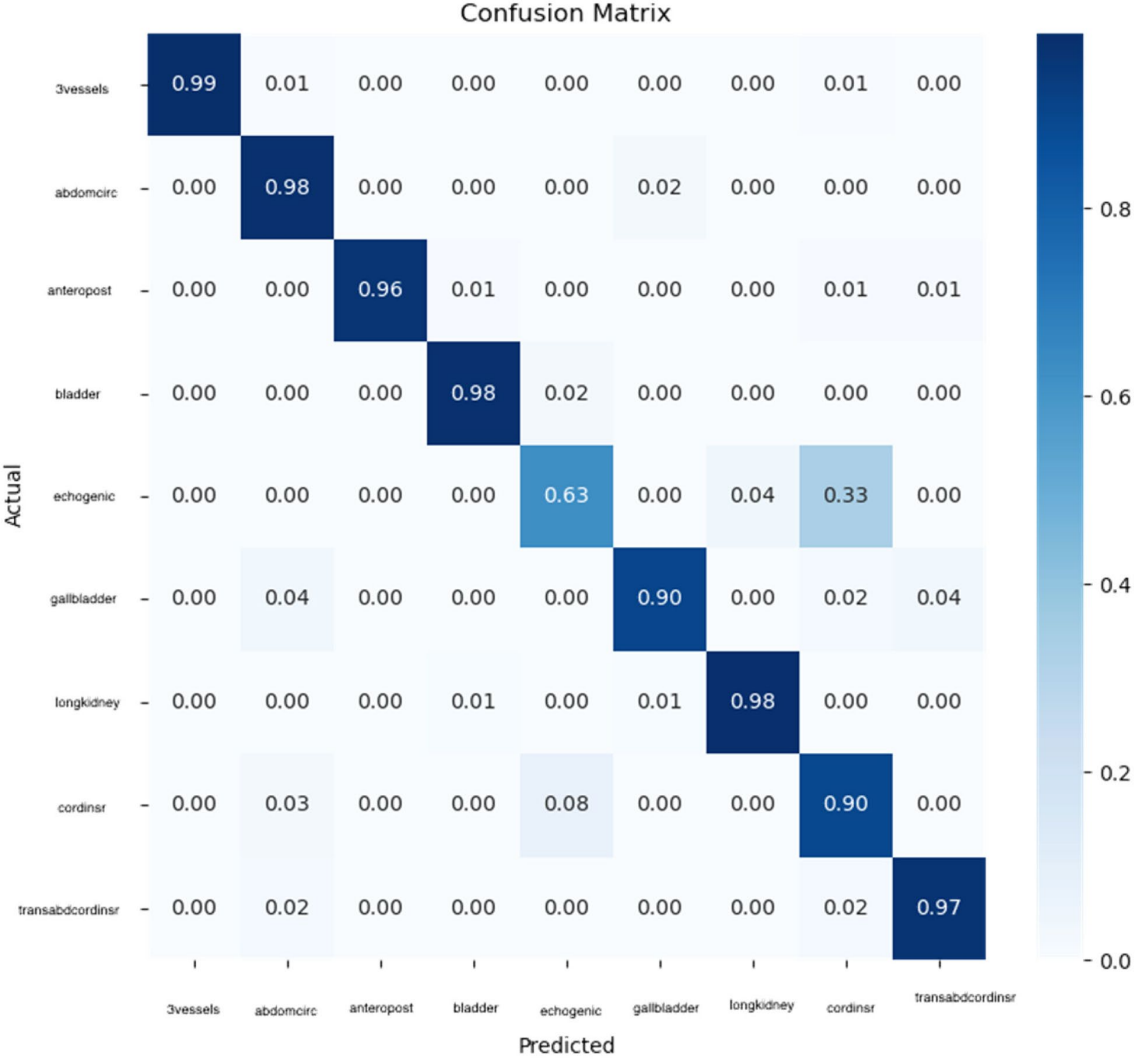
Confusion matrix for clustering view planes using the proposed framework

Besides the overall accuracy of the system, it is important to see the performance of differentiating between view planes per view plane. Hence, Fig. 5 presents the confusion matrix. We can see that most view planes are differentiated with accuracies, except echogenic. The clinical explanation for this fact is that the differences between the echogenic view plane and sagittal view plane is the presence of the wall and cord, otherwise the images are almost identical.

The next natural step before applying YOLO8 to segment the anatomical organs, is to ask ourselves why not use YOLO8 for view plane differentiation. Hence, we have made a quantitative comparison between our framework and YOLO8 performances on a hold-out test set. Taking into account the hypothesis of Wolpert and Macready, that there is ‘no-free-lunch’, we have considered our framework and YOLO8 as the ‘restaurants’, and the performance as the ‘dish’. In our case, the framework obtained better results when it came to view-plane differentiation of the fetal abdomen. Table 3 presents their performance in terms of ACA and SD.

**Table 3:** Comparison between the proposed framework and YOLO8 for image classification

Model	ACA	SD
Proposed framework	88.88	2.02
YOLO8	83.20	1.98

**Fig. 6:**
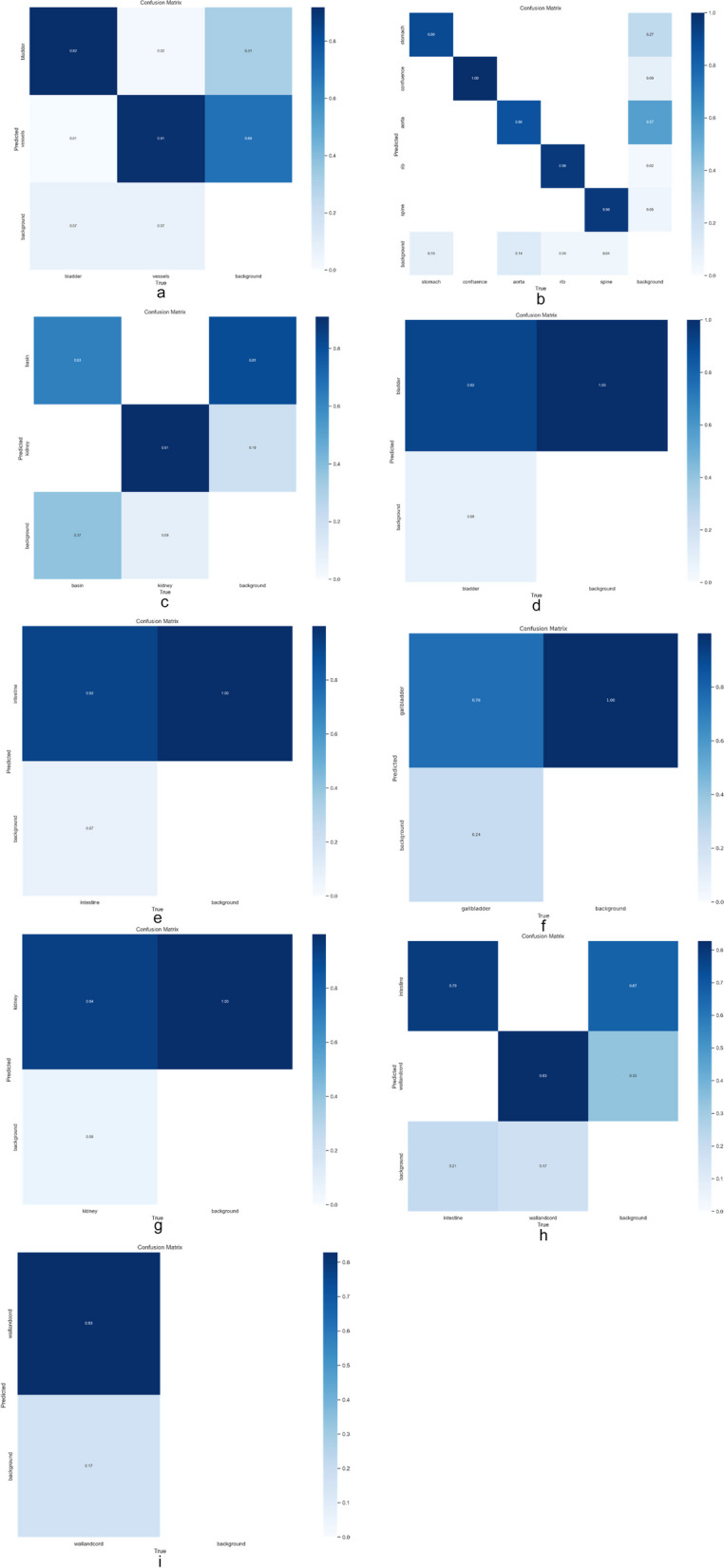
Confusion matrices for segmentation: 3 vessels plus bladder plane; abdominal circumference plane; anteroposterior kidney plane; bladder plane; echogenic plane; gallbladder plane; longitudinal kidney plane; cord insertion sagittal plane; transabdominal cord insertion plane

The final aim of our study was to apply YOLO8 to segment the anatomical organs, after clustering the view planes. Thus, after establishing which standard view plane we are dealing with, we applied YOLO8 and segmented the organs. Figure 6 shows the segmentation confusion matrices obtained for each view plane.

**Fig. 7:**
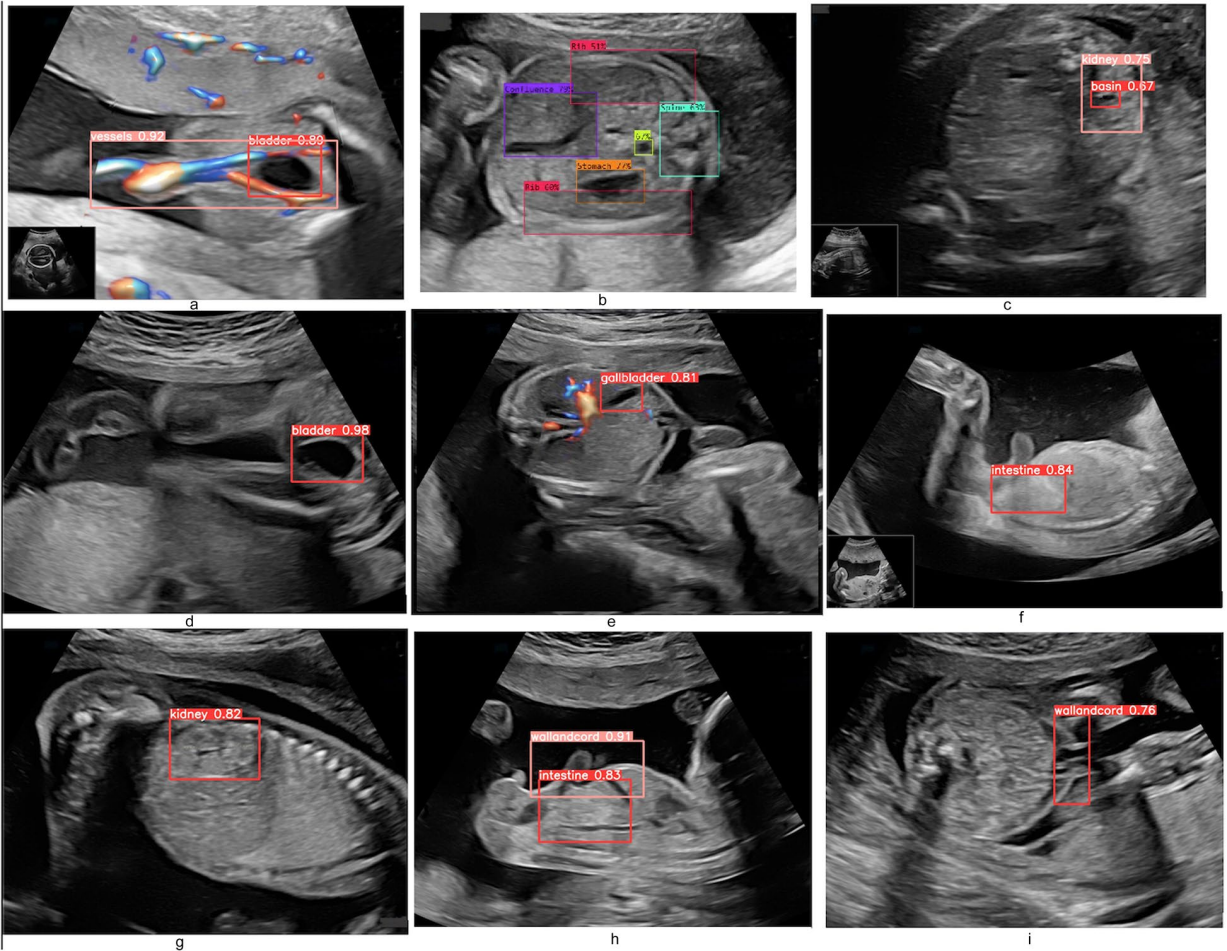
Automated segmentation of organs per view plane using Yolo: 3 vessels plus bladder plane; abdominal circumference plane anteroposterior kidney plane; bladder plane; echogenic plane; gallbladder plane; longitudinal kidney plane; cord insertion sagittal plane; transabdominal cord insertion plane

After training the AI model on the training data, we have quantitatively assessed the organ detection performance on real-time video ultrasounds (Fig. 7). We have measured the performance of the system using precision, recall, F1-score, Jaccard Index, and Probabilistic Rand Index. An important step in detecting the organs represents differentiating between the view planes, because many organs look the same, for example the bladder, the gallbladder and the stomach are represented by a black circle, hence without a proper plane detection, the system cannot differentiate between them. Therefore, the first step of the system, the clustering process, is needed.

In Table 4, we present the performance scores of YOLO8 for organ segmentation in real-time ultrasound movie. Additionally, the confusion matrix for the detection of each organ is presented in Fig. 6. The reported results for this experiment give us an indication of how the framework performs in a real scenario, that we observe in clinical practice. From Fig. 6 and Table 4, we see that the all the classes are differentiated very good, except the echogenic view plane. Regarding the organs, we have a very high detection rate, F1-score equal or above 0.9, for vessels, bladder, gallbladder, wall and cord (sagittal plane), a high detection rate, F1-score between 0.8 and 0.9, for stomach, intestines, kidney, intestines, and a fair detection rate, F1-score between 0.7 and 0.8, for rib and wall and cord (transversal plane). These accuracy levels are good in clinical practice, taking into account the fact that by breathing and moving the fetus changes the view planes instantly. Therefore, the most important part is to correctly differentiate between the view planes, before proceeding to segmenting the organs.

**Table 4:** Detailed classification per organ using real time ultrasound movie

**View plane**	**Organ**	**Precision**	**Recall**	**F1-score**	Jaccard Index	Probabilistic Rand Index
**3 vessels plus bladder**	Bladder	0.891	0.882	0.886	0.883	0.850
	Vessels	0.923	0.942	0.931	0.933	0.921
**Abdominal circumference**	Aorta	0.660	0.656	0.759	0.753	0.723
	Confluence	0.792	0.799	0.796	0794	0.764
	Rib	0.742	0.701	0.733	0.731	0.702
	Spine	0.755	0.748	0.754	0.746	0.721
	Stomach	0.861	0.872	0.870	0.869	0.812
**Bladder**	Bladder	0.988	0.976	0.978	0.979	0.934
**Echogenic**	Intestines	0.862	0.894	0.890	0.875	0.864
**Gallbladder**	Gallbladder	0.981	0.975	0.987	0.980	0.964
**Longitudinal kidney**	Kidney	0.832	0.876	0.871	0.846	0.852
**Cord insertion sagittal**	Intestines	0.833	0.803	0.822	0.832	0.819
	Wall and cord	0.911	0.906	0.909	0.901	0.899
**Transabdominal cord insertion**	Wall and cord	0.763	0.733	0.744	0.759	0.745

## Discussion

The above study was three-fold: *a*) to determine which DL algorithm is best suited to extract features from images for the clinical case in question, *b*) to determine which clustering technique groups best the extracted features, and *c*) to segment the anatomical organs in each classified view-plane using YOLO8. Having five state-of-the-art DL algorithms we have applied them on our dataset to extract the features from images. After benchmarking the DL algorithms, we have established that the best suited for this case is DenseNet121. We have applied three clustering techniques to group the feature vectors obtained after using DenseNet121. After benchmarking the clustering methods, we concluded that mean-shift clustering performed the best. We have used DenseNet121 and mean-shift clustering on a hold-out test and classified the images from one patient into the nine standard view planes of the fetal abdomen. Once we had the images classified, we applied YOLO8 and segmented the organs in each view plane. This AI powered decision support system proved to be efficient when applied onto this dataset. The AI system can be applied on other data after retraining.

The proposed AI powered decision support system is a promising step forward to signaling birth defects automatically. The system is able to detect correctly the view plane and the organs in that view plane. We proposed an intelligent tool that can assist unexperienced sonographers in performing second trimester fetal morphology scans. It should be noted that the system detected correctly the view planes and organs when applied on real time ultrasound movie. Future work will focus on another AI component of the system which will perform anomaly detection on the segmented organs. We have demonstrated the framework’s ability for a real-time localization of the abdomen view planes, and for a real-time localization of the organs in these planes.

Our approach is up-front and adaptable in different characteristics:

- We proposed a statistical benchmarking analysis that provided a good discrimination between the DL and clustering methods.
- The AI powered decision support system is easy to use and understand.
- The system is flexible and can be adapted to other clinical cases.

## Conclusions

We have proposed an AI powered decision support system that benchmarks different DL algorithms in the task of extracting features from 2D ultrasound scan images regarding second trimester fetal morphology. The extracted feature vectors are then clustered using three different clustering techniques. The results of the clustering process are once again statistically analyzed and the best performing method is used in the testing phase. We have compared our framework with YOLO8 on hold-out test images and demonstrated that our method outperforms YOLO8 in view plane classification on this particular dataset. Finally, YOLO8 was used to segment the organs on real-time ultrasound movie. The results of the systems are promising, and the design is straightforward.

Future work might lie in:

- Extending the study to other body zone: skull, limbs, etc.
- Using other DL and clustering algorithms.
- Designing an engine control unit that runs the AI system on a whole fetal morphology scan.

## Data Availability

The data will be made available upon request (email sbelciug@inf.ucv.ro).
